# State of the art in vulvar cancer imaging

**DOI:** 10.1590/0100-3984.2018.0072

**Published:** 2019

**Authors:** Maria Ana Serrado, Mariana Horta, Teresa Margarida Cunha

**Affiliations:** 1 Radiology Department, Hospital Central do Funchal, Funchal, Portugal.; 2 Radiology Department, Instituto Português de Oncologia de Lisboa Francisco Gentil, Lisboa, Portugal.

**Keywords:** Vulva, Vulvar neoplasms, Carcinoma, Lymph nodes, Radiology, Vulva, Neoplasias da vulva, Carcinoma, Gânglios linfáticos, Radiologia

## Abstract

Vulvar carcinoma is an uncommon tumor that predominantly affects postmenopausal women. Currently, there is no screening procedure for vulvar carcinoma; in most cases, it is diagnosed only when symptoms appear. The most widely used staging system is that developed by the International Federation of Gynecology and Obstetrics. Lymph node status is the most important prognostic factor. We searched the PubMed/Medline database to identify relevant English-language articles on vulvar cancer, with a special focus on its imaging evaluation. Magnetic resonance imaging is useful for local and nodal staging, as well as facilitating the planning of surgical interventions and radiotherapy. Computed tomography or positron-emission tomography/computed tomography can play an important role in nodal and distant disease assessment, whereas ultrasound is often used for image-guided biopsies. Imaging is pivotal for staging and treatment planning in vulvar carcinoma.

## Abstract (pt)

O carcinoma da vulva é um tumor incomum que afeta predominantemente mulheres em menopausa. Atualmente, não existe um teste de rastreio para o carcinoma da vulva, e a maioria dos casos é diagnosticada com o aparecimento de sintomas. O sistema de estadiamento mais frequentemente utilizado é o da International Federation of Gynecology and Obstetrics. O fator prognóstico mais importante é o estadiamento ganglionar. Neste trabalho foram efetuadas pesquisas na base de dados PubMed/Medline, considerando-se textos pertinentes em língua inglesa sobre carcinoma da vulva, com especial ênfase na avaliação radiológica. A ressonância magnética é útil para a avaliação local e ganglionar e pode ajudar no planejamento cirúrgico e/ou de radioterapia; a tomografia computadorizada ou a tomografia com emissão de pósitrons/tomografia computadorizada pode ser importante na apreciação ganglionar e na doença a distância; e a ultrassonografia é, por vezes, utilizada para orientar procedimentos de intervenção, como a biópsia. A radiologia é fundamental no estadiamento e planejamento do tratamento do carcinoma da vulva.

## INTRODUCTION

Carcinoma of the vulva is an uncommon tumor, accounting for only approximately 4% of gynecologic malignancies. In the United States, there were an estimated 6190 new cases of vulvar cancer and 1200 deaths associated with the disease in 2018^(^^1^^)^. It predominantly affects postmenopausal women, its incidence increasing with age^(^^2^^)^. Currently, there is no screening procedure for vulvar carcinoma. However, patients with a history of cervical or vaginal cancer, vulvar intraepithelial neoplasia, or lichen sclerosus should be kept under regular surveillance^(^^2^^)^.

In most cases, vulvar carcinoma is symptomatic, presenting as a painful vulvar lump or ulcer, pruritus, bleeding, and discharge. When there are metastases to the inguinal lymph nodes, a lump in the groin can be reported. Occasionally, vulvar carcinoma is asymptomatic^(^^2^^)^. It is confined to the primary site in 59% of the cases, spreads to regional lymph nodes in 30%, and causes distant disease in 6%. The 5-year relative survival rate is linked to the stage at diagnosis. The 5-year survival rate is 86% for localized disease, 53% for regional spread, and 19% for distant disease^(^^3^^)^. The most important prognostic factor is lymph node status. Survival is impaired in patients with positive lymph nodes; 88% of node-negative patients are free of disease within two years. Of the patients with one, two, or more than two positive lymph nodes, 60%, 43%, and 29%, respectively, are free of disease within two years^(^^4^^)^. The management of vulvar carcinoma must therefore be individualized.

The aim of this study was to review the imaging aspects of vulvar carcinoma, its staging, and the influence of imaging findings on treatment planning. We searched the PubMed/Medline database, using the following search terms: vulvar cancer; vulvar carcinoma; gynecologic malignancies; ultrasound; computed tomography (CT); magnetic resonance imaging (MRI); and positron-emission tomography/computed tomography (PET/CT). We selected relevant English-language papers on vulvar cancer, with a special focus on its imaging evaluation.

## VULVAR ANATOMY, VULVAR CARCINOMA, AND TUMOR SPREAD

**Figure 1:**
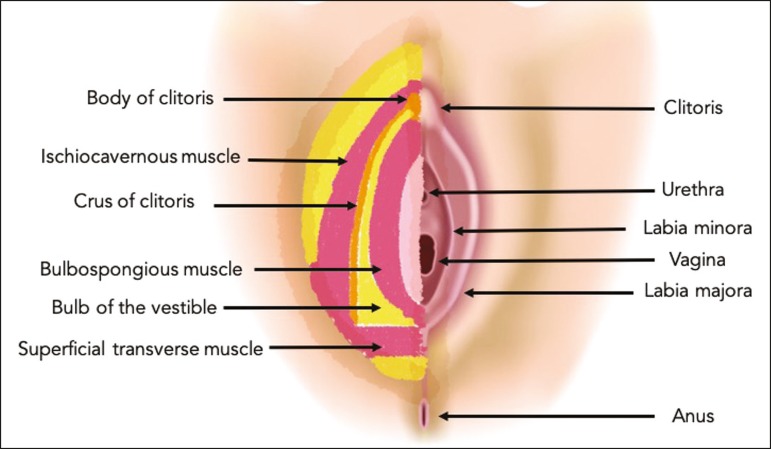
Schematic representation of the superficial and deep structures of the vulva.

The vulva is bounded anteriorly by the symphysis pubis, posteriorly by the anal sphincter, superiorly by the urogenital diaphragm, and laterally by the ischial tuberosity^(^^5^^,^^6^^)^. As depicted in Figure 1, it is composed of superficial structures (including the labia majora and minora), as well as the vestibule of the vagina, opening of the urethra, clitoral glans, and clitoral body; the bulbospongiosus and ischiocavernosus muscles; and the vestibular bulbs and the crura of the clitoris. The clitoris is composed of the glans, body, and crura (right and left), which are in turn composed of the corpora cavernosa and covered by the ischiocavernosus muscle. The vestibular bulb is a venous plexus surrounded by fascia and covered by the bulbospongiosus muscle^(^^5^^)^.

The vulva is irrigated via branches of the external and internal pudendal arteries^(^^6^^)^. The venous drainage is mainly via the (external and internal) pudendal veins, perineal vein, and deep posterior vein of the clitoris^(^^5^^)^. The lymphatic drainage from the vulva is primarily via the superficial inguinal nodes, although the deep inguinal nodes are also involved^(^^6^^)^. The superficial inguinal nodes are located deeper than the inguinal ring and can be divided into three groups: medial, intermediate, and lateral. The deep inguinal nodes lie medial to the femoral vein^(^^7^^)^. The subsequent drainage from those nodes is via the external iliac and pelvic nodes^(^^5^^,^^8^^,^^9^^)^.

Vulvar carcinomas can be divided into those that are related to human papillomavirus infection and those that are not. The former lead to squamous cell carcinoma in younger women and account for 20% of all cases of invasive disease. The latter lead to squamous cell carcinoma in older patients and account for 80% of all cases of invasive disease^(^^10^^)^.

Nearly 70% of vulvar carcinomas involve the labia majora and minora. In 15-20% of cases, the lesion involves the clitoris. In approximately 10% of cases, the lesion is so extensive that the primary location cannot be identified. Multifocal lesions occur in 5% of cases^(^^5^^)^.

**Figure 2:**
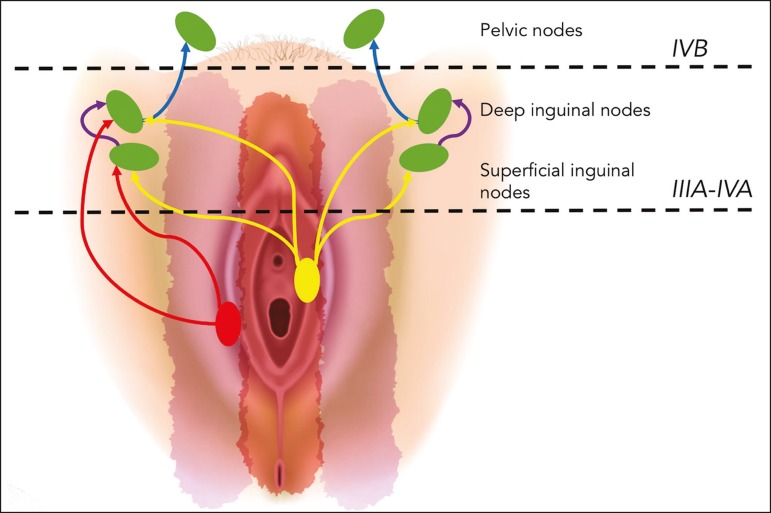
Schematic representation of lymphatic drainage of carcinoma of the vulva.

Lateral vulvar carcinomas drain to the ipsilateral inguinal lymph nodes. Because of the rich lymphatic network across the midline of the vulva, midline lesions and lesions within 1 cm of the midline can drain bilaterally^(^^5^^,^^7^^,^^8^^)^. The deep inguinal lymph nodes can be infiltrated by tumor cells, although the superficial inguinal lymph nodes are not^(^^7^^)^. As can be seen in Figure 2, the pelvic lymph nodes are rarely involved unless the ipsilateral inguinal lymph nodes are as well^(^^5^^,^^7^^,^^8^^)^.

In rare cases, vulvar carcinoma can spread to the pelvic lymph nodes via the internal pudendal chain and internal iliac chain, as direct drainage from the midline^(^^5^^,^^8^^)^. If there is obstruction of the typical lymphatic drainage, the tumor can spread via the subcutaneous and dermal lymphatic systems of the upper thigh and lower abdomen^(^^5^^,^^8^^)^. If the tumor has invaded the vagina, bladder, or anus (above the dentate line), it can spread to the obturator or internal iliac lymph nodes^(^^5^^)^. Distant metastases are rare if the disease is diagnosed in its early stages^(^^11^^)^.

## THE ROLE OF IMAGING

### Imaging appearance of the vulva

Due to its excellent soft tissue resolution, MRI is the ideal imaging modality for assessing vulvar anatomy. The normal vulva has low to intermediate signal intensity on T1-weighted imaging (T1WI), whereas the signal intensity on T2-weighted imaging (T2WI) is slightly higher^(^^6^^)^.

Even with intravenous contrast agent administration, CT is limited in its ability to depict the vulva. On CT, the vulva appears as a triangular soft tissue structure^(^^5^^)^.

### Imaging modalities

#### Ultrasound and ultrasound-guided fine-needle aspiration cytology

The role of ultrasound and fine-needle aspiration cytology (FNAC) in the evaluation of nodal status in vulvar cancer has been subject of several studies. When used in isolation, the reported sensitivity of ultrasound ranges from 76% to 100% and its reported specificity ranges from 69% to 91%^(^^12^^-^^16^^)^. That wide variation can be attributed to a variety of factors related to the lymph node in question, including the short-axis diameter, the ratio of long- to short-axis diameter, an irregular shape, and the absence of a fatty hilum, as well as the overall attenuation and peripheral vascularization. It has been suggested that greater peripheral vascularity on color Doppler, especially on power Doppler, is indicative of metastatic nodes. A spectral waveform showing a high resistive index can further strengthen that hypothesis^(^^15^^)^.

Moskovic et al.^(^^13^^)^ studied the diagnostic performance of FNAC in comparison with that of the combination of ultrasound and FNAC. The authors found that the use of FNAC alone had a sensitivity and specificity of 58% and 100%, respectively, and that combining ultrasound with FNAC increased the sensitivity to 83% but reduced the specificity to 82%. In a subsequent study conducted at the same institution and evaluating the same parameters, the accuracy of that combination was shown to have improved over time, reaching a sensitivity of 93% and a specificity of 100%^(^^15^^)^. Consequently, some authors have suggested that the combination of ultrasound and FNAC can prevent unnecessary inguinal lymph node dissection^(^^15^^)^. However, the use of ultrasound alone cannot replace surgical lymph node staging^(^^16^^)^.

#### MRI

**Figure 3:**
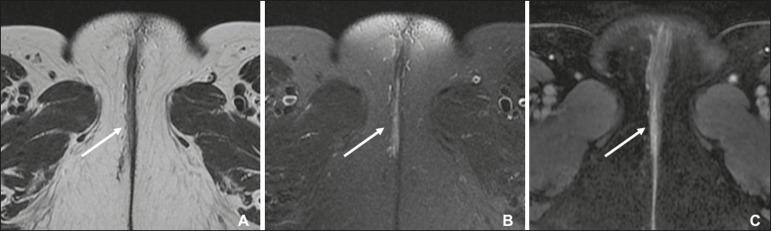
FIGO stage IB vulvar carcinoma. **A:** Axial T2WI showing a right-sided vulvar lesion with intermediate signal intensity (arrow). **B:** Axial T2WI with fat suppression better delineated the tumor (arrow). **C:** Contrast-enhanced axial T1WI with fat suppression, showing the same tumor (arrow). Pathology, at surgery, revealed a 37 mm tumor with 5 mm of invasion, confirming the diagnosis of stage IB disease.

Patient preparation is important for obtaining optimal results with MRI. Fasting for 4-6 hours before the examination, anti-peristaltic agent administration, bladder voiding, and vaginal distention with ultrasound gel may all be considered. The standard MRI protocol for the evaluation of vulvar lesions includes axial T1-weighted fast spin-echo images with a large field of view; axial and coronal high-resolution T2-weighted fast spin-echo images; and sagittal T1WI^(^^7^^)^. T2WI with fat suppression may show the tumor better than do images without fat suppression^(^^7^^,^^9^^,^^17^^)^, given that the perineal region is rich in fat, that tumors with intermediate signal intensity on T2WI (Figure 3A) are more difficult to visualize, and that the intrinsic signal intensity of the tumor is associated with necrosis (Figure 3B). Dynamic contrast-enhanced MRI can aid in the evaluation of small tumors and of involvement of the urethra, anus, and vagina^(^^7^^,^^9^^)^, as shown in Figure 3C. Diffusion-weighted imaging can also be useful for delineating primary masses and lymph node metastases^(^^5^^,^^9^^)^.

The assessment of primary vulvar lesions is best achieved by MRI. Sohaib et al.^(^^18^^)^ reported that the MRI staging of vulvar cancers was correct in 70% of cases. Kataoka et al.^(^^17^^)^ studied the accuracy of unenhanced and contrast-enhanced MRI in determining the size and stage of vulvar cancers. In the evaluation of lesion size, unenhanced MRI was found to have an accuracy of 86% for primary vulvar cancers and 72% for recurrent vulvar cancers, resulting in an overall accuracy of 83%. The use of contrast did not change the accuracy of MRI in the evaluation of the size of primary vulvar cancers, although it did increase that accuracy (to 80%) in recurrent vulvar cancers. For the staging of primary vulvar cancer, the reported accuracy of unenhanced MRI is 69%, whereas that of contrast-enhanced MRI is 85%^(^^17^^)^. MRI is also the best imaging method of determining the involvement of adjacent structures, which is pivotal for the surgical planning.

Some studies have used MRI to evaluate lymph node metastases in vulvar cancer. Those studies have reported widely varying sensitivities and specificities, ranging from 40% to 89% and from 81% to 100%, respectively^(^^17^^-^^21^^)^. In addition, several criteria have been proposed as being the most accurate in identifying suspicious lymph nodes. Although the short-axis diameter is the most commonly used criterion, its sensitivity is low, ranging from 40% to 50%, depending on the location of the lymph node and the cut-off value employed^(^^18^^)^.

Hawnaur et al.^(^^19^^)^ suggested that the most useful single criterion is that of lymph node contour irregularity. It has also been suggested that the ratio of long- to short-axis diameter is a good quantitative marker for diagnosing lymph node metastases, with a sensitivity of 85% and a specificity of 81%^(^^17^^)^. Among individual criteria, lymph node necrosis has been shown to have the highest specificity, although it has low sensitivity^(^^17^^)^. Bipat et al.^(^^20^^)^ demonstrated that, in the evaluation of several criteria, such as short-axis diameter, aspect, margin, and ratio of long- to short-axis diameter, the interobserver agreement was good (kappa = 0.62). False-positive results can be related to reactive changes, particularly when the MRI is performed soon after a diagnostic vulvar biopsy^(^^21^^)^.

#### CT

In suspected cases of vulvar cancer, CT should be performed with oral, intravenous, and rectal contrast administration. Images are usually obtained with 2.5-mm axial slice reconstructions, including the upper thighs to cover the entire extent of the involvement^(^^7^^)^. However, CT is of limited usefulness in the evaluation of primary vulvar lesions. It is not routinely recommended for the detection of inguinal lymph nodes, for which it is reported to have a sensitivity of 58-60%, a specificity of 75-90%, a positive predictive value of 38-58%, and a negative predictive value of 75-96%^(^^12^^,^^22^^)^. Preoperative CT has been shown to have no influence on the treatment strategy^(^^12^^,^^22^^)^. Therefore, CT may play a role only when there is clinical evidence of diseased inguinal lymph nodes-in order to assess the proximity of such nodes to the femoral vessels and to evaluate the pelvic lymph nodes-or when there is surgically proven inguinal lymph node involvement-in order to plan the postoperative treatment^(^^12^^,^^22^^)^.

Because a short-axis diameter of ≥ 1 cm is the standard criterion to identify metastatic lymph nodes, metastatic lymph nodes < 1 cm may be missed on CT^(^^7^^)^. In a study conducted by Lin et al.^(^^23^^)^, CT and MRI were both found to be significantly more efficacious than was ^18^F-fluorodeoxyglucose PET/CT (^18^F-FDG PET/CT) in detecting pelvic lymph node involvement and distant metastases. However, the authors stated that the high sensitivity and negative predictive value of ^18^F-FDG PET/CT can be reassuring after suspicious lymph nodes have been identified on CT or MRI.

#### ^18^F-FDG PET/CT

In ^18^F-FDG PET/CT, the imaging is performed 60-90 min after intravenous administration of ^18^F-FDG. The body is scanned, in the caudocranial direction, from the upper thighs to the skull vertex. Patients are required to empty their bladder before imaging. The studies are usually performed on an integrated PET/CT scanner. Unenhanced axial CT is performed for attenuation correction and diagnosis, thereafter being reconstructed to match the slice thickness of the PET images^(^^7^^)^.

The accuracy of ^18^F-FDG PET/CT in detecting primary vulvar lesions is 100%^(^^24^^)^. However, like CT, it plays a limited role in the assessment of the extent of the tumor. Several studies have investigated the role of PET and ^18^F-FDG PET/CT in the evaluation of inguinal lymph nodes, reporting a wide range of diagnostic performance levels^(^^23^^-^^27^^)^. Sensitivities ranging from 50% to 100% and specificities ranging from 67% to 100% have been reported. The majority of such studies state that ^18^F-FDG PET/CT is not indicated in nodal staging, because of its low sensitivity^(^^25^^-^^27^^)^. The ideal cut-off of maximum standardized uptake value (SUV_max_) is not well established in the literature. The reported range for the mean SUV_max_ of metastatic inguinal lymph nodes is from 6.1 to 11.0^(^^23^^,^^26^^)^. ^18^F-FDG PET/CT has limited value in the evaluation of lymph node metastases < 5 mm. It can also produce false-negative results in necrotic lymph nodes and false-positive results in inflammatory lymph nodes^(^^7^^,^^24^^)^. The 100% negative predictive value of ^18^F-FDG PET/CT can be supportive when pelvic lymph node involvement or distant metastases are suspected on CT or MRI^(^^23^^)^.

## STAGING SYSTEM, IMAGING FINDINGS, AND TREATMENT

The most widely used staging system is that developed by the International Federation of Gynecology and Obstetrics (FIGO), which was revised in 2009^(^^28^^)^. As previously reported^(^^2^^,^^29^^)^, the 2009 revision of the FIGO staging system corrected several previous limitations, in the following ways (Table 1): by combining stages IB and II into a new stage IB; by dividing stage III into the new stages II and III; by stating that all cases with positive inguinal lymph nodes should now be classified as stage III; and by no longer considering bilateral inguinal disease. Those changes have been validated in several studies^(^^30^^-^^32^^)^.

**Table 1:** FIGO staging for carcinoma of the vulva, 2009 revision.

Stage	Definition
I	Tumor confined to the vulva.
IA	Lesions ≤ 2 cm in size, confined to the vulva or perineum and with stromal invasion ≤ 1 mm, no nodal metastasis.
IB	Lesions > 2 cm or with stromal invasion < 1 mm, confined to the vulva or perineum, with negative lymph nodes.
II	Tumor of any size extending to adjacent perineal structures (lower 1/3 of the urethra, lower 1/3 of the vagina, or anus) with negative lymph nodes.
III	Tumor of any size, with or without extension to adjacent perineal structures (lower 1/3 of the urethra, lower 1/3 of the vagina, or anus), with positive inguinofemoral lymph nodes.
IIIA	i) 1 Lymph node metastasis ≥ 5 mm; or ii) 1-2 lymph node metastases < 5 mm.
IIIB	i) ≥ 2 Lymph node metastases ≥ 5 mm; or ii) ≥ 3 lymph node metastases < 5 mm.
IIIC	Positive lymph nodes with extracapsular spread.
IV	Tumor invades other regional structures (upper 2/3 of the urethra or upper 2/3 of the vagina) or distant structures.
IVA	i) Tumor invasion of the upper urethra, vaginal mucosa, bladder mucosa, rectal mucosa, or pelvic bone; or (ii) fixed or ulcerated inguinofemoral lymph nodes.
IVB	Distant metastasis to any site, including the pelvic lymph nodes.

### Stages I and II-localized disease

Stage IA disease is defined as a tumor ≤ 2 cm with stromal invasion ≤ 1 mm. Stage IB is defined as a tumor > 2 cm with stromal invasion > 1 mm. In both stages, there is no nodal involvement, invasion of adjacent structures, or distant disease. Stage II is defined as a tumor of any size with invasion of adjacent perineal structures.

#### Imaging findings

**Figure 4:**
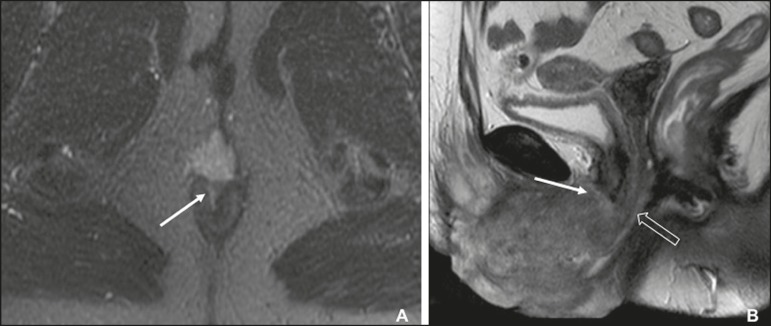
FIGO stage II vulvar carcinoma. **A:** Axial oblique T2WI with fat saturation, clarifying the extension to the anal wall (arrow). The patient was treated with chemoradiotherapy and showed no clinical evidence of disease at 6 months of follow-up. **B:** Sagittal T2WI showing a vulvar tumor extending to the lower thirds of the urethra (arrow) and vagina (open arrow).

The role of imaging is limited in stages IA, IB, and II. Vulvar carcinoma is depicted as a solid mass with low signal intensity on T1WI and intermediate to high signal intensity on T2WI. On dynamic contrast-enhanced sequences (Figure 3), there is contrast enhancement in the early arterial phase^(^^7^^)^. Disruption of the target appearance of the urethra on T2WI and interruption of the low signal intensity on T2WI of the vaginal wall (Figure 4A) or anal sphincter by an intermediate to high signal intensity tumor on T2WI (Figure 4B) are suggestive of invasion^(^^9^^)^. On CT images, a vulvar tumor appears as an area of vulvar thickening or a vulvar mass^(^^7^^,^^9^^)^. On ^18^F-FDG PET/CT, the primary tumor may appear as an area of focal hypermetabolism^(^^7^^)^.

#### Treatment

In stages IA, IB, and II, the first-line treatment is surgery. For stage IA tumors, wide or radical local resection is indicated^(^^33^^,^^34^^)^. Inguinofemoral lymph node evaluation is not necessary, given the low risk of metastases to those lymph nodes^(^^33^^,^^34^^)^. For stages IB and II, the primary treatment is determined by the tumor location. Patients with lateral lesions (i.e., lesions located > 1 cm from the vulvar midline) should undergo radical local resection or modified radical vulvectomy, together with evaluation of the ipsilateral inguinal lymph node. Patients with midline vulvar lesions should undergo radical local resection or modified radical vulvectomy and evaluation of the right and left inguinal lymph nodes^(^^33^^,^^34^^)^. If the distal urethra or vagina is involved, resection with clear margins is an option. If the anus is involved, preoperative radiotherapy, with or without concurrent chemotherapy, may be considered, in order to spare the patient a colostomy^(^^2^^)^.

Inguinal lymph node evaluation may be performed through sentinel node biopsy or inguinofemoral lymph node dissection. The sentinel lymph node procedure is recommended for unifocal tumors < 4 cm in patients without clinical or radiological evidence of lymph node disease. Inguinofemoral lymphadenectomy is recommended for tumors ≥ 4 cm and in cases of multifocal disease^(^^34^^)^. Inguinal lymphadenectomy is indicated when sentinel lymph nodes are not detected^(^^33^^,^^34^^)^. Contralateral inguinofemoral lymph node dissection may be performed when there is metastases to the ipsilateral nodes^(^^34^^)^.

The need for adjuvant therapy is determined by the nodal and primary tumor pathology observed during surgery. In stages IA, IB, or II with negative lymph node status, observation is usually adequate^(^^33^^)^. In stages IB or II with positive lymph node status, adjuvant therapy includes external beam radiotherapy (EBRT), with or without concurrent chemotherapy; or EBRT, with or without concurrent chemotherapy, and completion inguinofemoral lymph node dissection, if not previously done. If the surgical margins are negative, observation is indicated. Conversely, if surgical margins are close (> 8 mm) or positive, another resection should be attempted. In cases of persistent positive margins or in patients who are not candidates for a second resection, EBRT is recommended^(^^33^^)^.

### Stages III and IVA-locally advanced or regional disease

**Figure 5:**
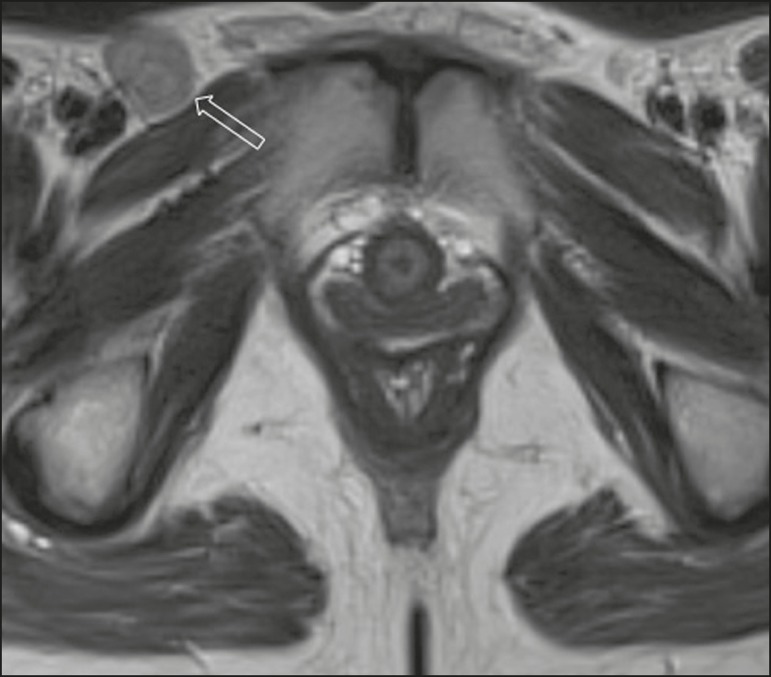
FIGO stage III vulvar carcinoma. Axial T2WI showing an enlarged right inguinal lymph node (open arrow). Fine needle aspiration cytology of the lymph node confirmed the presence of tumor cells within the node.

Stage III vulvar carcinoma is defined as inguinal node involvement (Figure 5). When it affects one or two inguinal lymph nodes < 5 mm or a single inguinal lymph node ≥ 5 mm, it is classified as stage IIIA. Stage IIIB is defined as involvement of two or more inguinal lymph nodes ≥ 5 mm or three or more < 5 mm. Stage IIIC is defined as positive lymph nodes with extracapsular spread.

Stage IVA vulvar carcinoma is defined as a tumor that is fixed to or involves the pelvic bones; that invades the upper urethral or vaginal mucosa, bladder or rectum; or that results in fixed or ulcerated inguinofemoral lymph nodes.

### Stage IVB-metastatic disease

Stage IVB vulvar carcinoma is defined as distant metastatic disease. It involves regional metastases to inguinal lymph nodes.

#### Imaging findings

**Figure 6:**
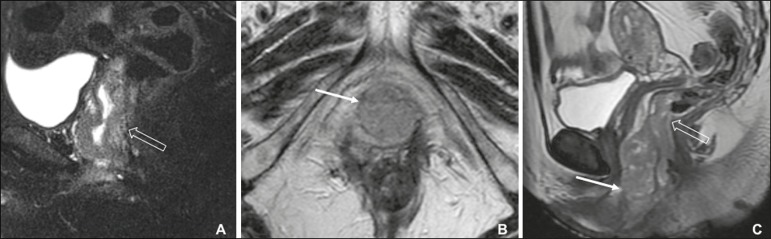
FIGO stage IVA vulvar carcinoma. **A:** Sagittal T2WI with fat saturation showing involvement of the upper two thirds of the vagina (open arrow). The patient was treated with chemoradiotherapy and showed no clinical evidence of disease at two years of follow-up. **B:** Axial T2WI showing a central vulvar lesion with intermediate signal intensity involving the upper two thirds of the urethra (arrow). The patient was treated exclusively with radiotherapy, being ineligible for concurrent chemotherapy because of a low performance status. **C:** Sagittal T2WI showing a vulvar lesion with intermediate signal intensity (arrow) extending to the rectum (open arrow). The patient was treated with chemoradiotherapy and had a recurrence at 27 months after the initial diagnosis.

On cross-sectional imaging, lymph nodes are usually considered suspicious when the short-axis diameter is > 1 cm, which is the criterion most commonly used for MRI. The morphology, contour, attenuation, and signal intensity are ancillary findings that can facilitate the diagnosis of metastatic lymph nodes. The low signal intensity of the upper two thirds of the vagina can be disrupted by an intermediate-signal tumor on T2WI (Figure 6A), as can the normal target appearance of the upper two thirds of the urethra (Figure 6B) and the normal high signal intensity of the bladder or rectal mucosa (Figure 6C). For CT, the parameters used for lymph node evaluation are the same as those used for MRI. On ^18^F-FDG PET/CT, metastatic lymph nodes are FDG avid^(^^7^^)^.

#### Treatment

All patients with stage III or IVA vulvar carcinoma should receive EBRT with concurrent chemotherapy. Patients with imaging-positive lymph nodes should undergo inguinofemoral dissection or FNAC^(^^33^^)^. If inguinofemoral dissection reveals no metastatic lymph nodes, the patient should receive radiotherapy covering the primary tumor (as well as the groin in selected cases) with concurrent chemotherapy should be done. If inguinofemoral dissection is not performed or is positive, radiotherapy with concurrent chemotherapy, with radiotherapy coverage of the primary tumor, groin and pelvis is indicated^(^^33^^)^.

The need for adjuvant therapy is based on the response to chemoradiotherapy. When there is no clinical evidence of residual tumor, observation is recommended. Patients with residual tumor should be considered for resection and patients with unresectable residual disease are candidates for additional EBRT, with or without systemic therapy^(^^33^^)^. For distant metastases, systemic (palliative) treatment may be considered on a case-by-case basis^(^^33^^,^^34^^)^.

## FOLLOW-UP AND RECURRENT DISEASE

**Figure 7:**
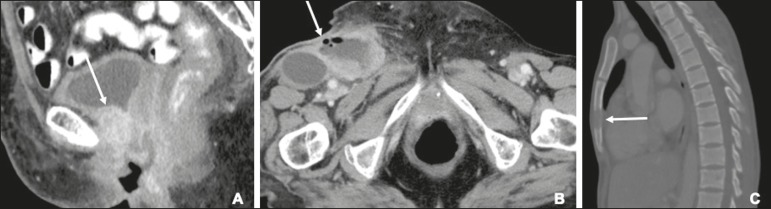
Recurrence of vulvar carcinoma. **A:** Contrast-enhanced sagittal CT showing extension to the floor of the bladder (arrow) at eight years after the initial diagnosis and four years after a first recurrence. **B:** Contrast-enhanced axial CT showing a necrotic right inguinal lymph node (arrow) extending to the skin, at 8 months after modified radical vulvectomy and bilateral inguinofemoral lymphadenectomy. **C:** Contrast-enhanced axial CT, with a bone-window setting, showing lytic metastases in the sternum (arrow). The patient was treated with chemoradiotherapy and had a recurrence at 13 months after the initial diagnosis.

Recurrent disease after vulvar carcinoma is common, occurring in 25-37% of cases^(^^11^^,^^35^^)^. The most common sites of recurrence are the perineal/vulvar region (Figure 7A), seen in 53-69% of patients with recurrence; the inguinal region (Figure 7B), seen in 19-24%; the pelvic region, seen in 6-8%; distant sites, seen in 4-8%; and multiple locations, seen in 8-14%. Distant metastases usually involve the following sites^(^^11^^,^^35^^)^: the lung, in 45-53% of cases; the liver, in 29%; soft tissue and lymphatic systems, in 15-27%; bone (Figure 7C), in 20-25%; and the skin, in 20%.

Isolated perineal recurrences are more common in stage I patients, whereas distant and multiple recurrences are more common in patients with advanced-stage disease. In addition, more than half of the isolated perineal recurrences occur in patients with negative inguinal lymph nodes, whereas 40% of multiple recurrences occur in patients with more than three positive lymph nodes^(^^35^^)^.

Perineal recurrences appear with an equal distribution in the first year after diagnosis and in the following years. Recurrences at other locations appear predominantly within the first two years. In patients with perineal recurrence, the 5-year survival rate is 60%, being considerably lower in those with pelvic or distant metastases. The corollary of these observations is that lymph node metastasis is a predictor of earlier recurrence, which is considered to confer a poorer prognosis^(^^35^^)^.

The cornerstone of the follow-up of patients with vulvar carcinoma is history taking and clinical examination, with careful examination of the vulva and groin regions, because of the propensity for local recurrence^(^^36^^,^^37^^)^. The use of imaging methods for the detection of asymptomatic distant disease is of unproven benefit and is unlikely to lead to improvements in survival, given the poor prognosis and relative ineffectiveness of salvage therapies in patients with distant recurrence^(^^36^^,^^37^^)^.

The available data do not support the routine use of imaging of the groin in the postoperative follow-up of patients with vulvar carcinoma. However, at 10-12 weeks after definitive chemoradiotherapy, CT or ^18^F-FDG PET/CT should be performed in order to document complete remission^(^^34^^)^.

In cases of vulvar recurrence, radical local excision with inguinofemoral lymphadenectomy is recommended, assuming that it was not previously performed or if only sentinel node dissection was performed. The indications for adjuvant therapy are comparable to those for the treatment of the primary disease^(^^33^^,^^34^^)^. When surgical treatment is not possible, EBRT, with or without brachytherapy or concurrent chemotherapy, is recommended^(^^33^^)^.

For nodal recurrence of vulvar carcinoma, the preferred treatment is radical excision. In radiotherapy-naïve patients, surgery may be followed by EBRT, with or without concurrent chemotherapy. Definitive chemoradiotherapy is appropriate when surgical treatment is not feasible^(^^33^^,^^34^^)^.

## CONCLUSION

Although the role of imaging methods in common gynecological malignancies is well established, little has been published about its role in vulvar carcinoma. In brief, MRI is useful for local and pelvic nodal staging, as well as informing the planning of surgical interventions and radiotherapy, whereas CT or ^18^F-FDG PET/CT can play an important role in the assessment of distant disease and ultrasound is often used for image-guided biopsy procedures.
